# Supplementary material for: Chromatin accessibility mapping of the striatum identifies tyrosine kinase FYN as a therapeutic target for heroin use disorder
Source: Nat Commun. 2020 Sep 14;11:4634. doi: 10.1038/s41467-020-18114-3 (PMC7490718; doi:10.1038/s41467-020-18114-3)
Supplement: Supplementary file 1 — Supplementary Information [file 41467_2020_18114_MOESM1_ESM.pdf]

Chromatin accessibility mapping of the striatum identifies tyrosine kinase FYN as a therapeutic target for heroin use disorder

Egervari et al.

Supplementary Figure 1

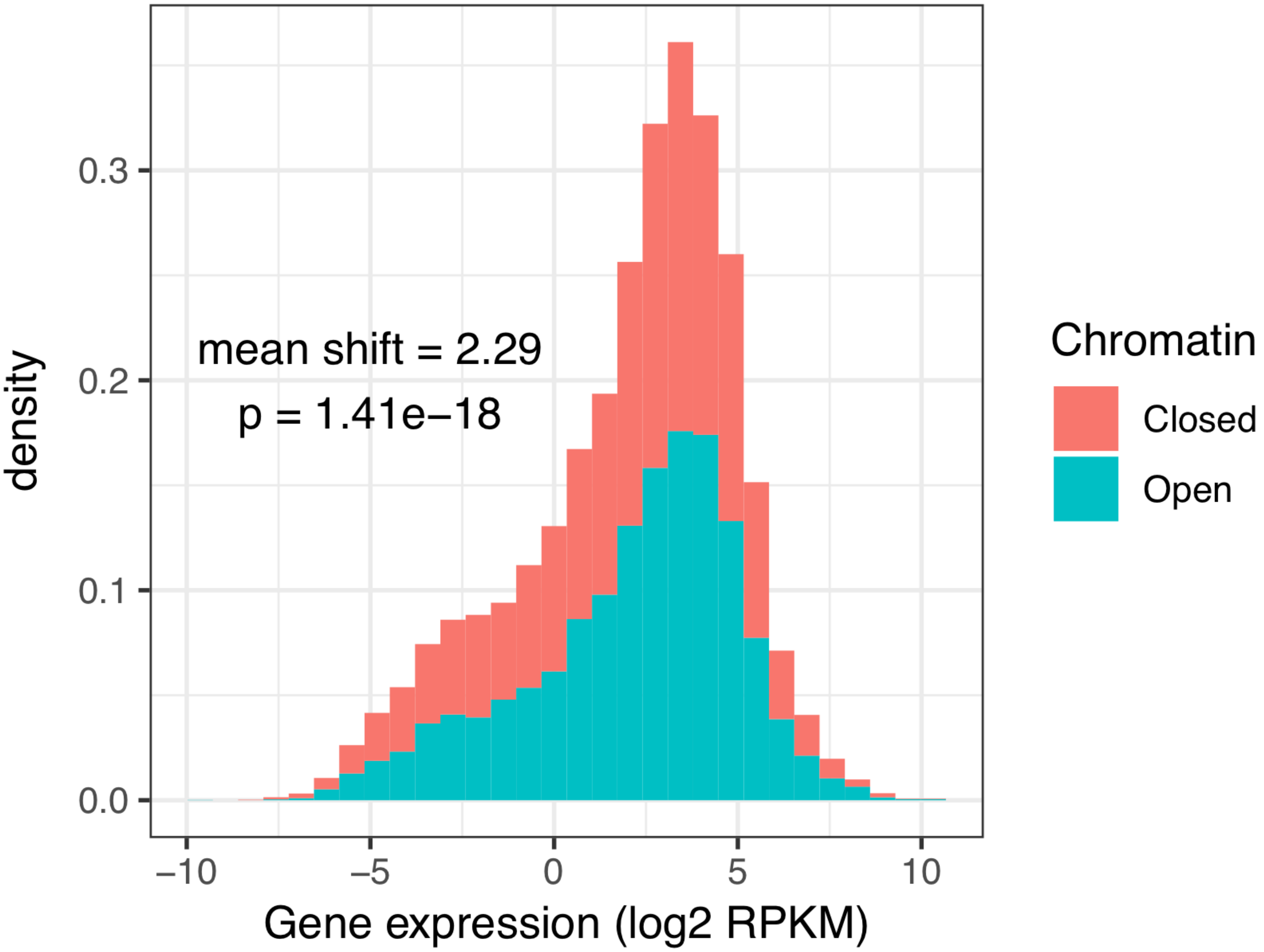

Supplementary Figure 1.
ATAC signal correlates to gene expression in the post-mortem human brain. Open genes [as defined by the presence of ATAC peak(s) within 10 kb of the TSS] have significantly higher gene expression (data from CommonMind Consortium) compared to closed genes (P value from Mann-Whitney U test). RPKM: reads per kilobase of transcript, per million mapped reads

Supplementary Figure 2

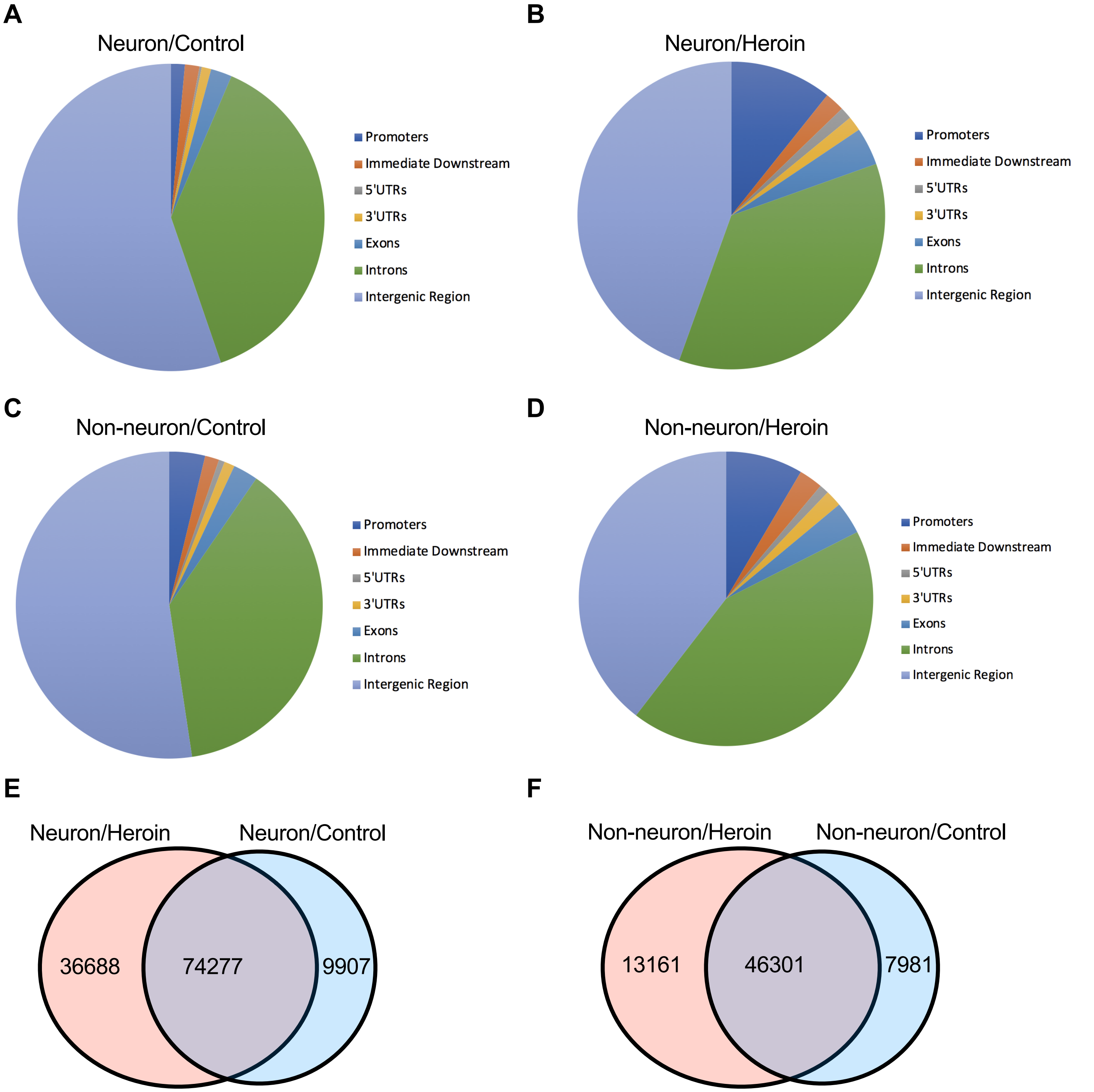

Supplementary Figure 2.
Re-distribution of chromatin accessibility by heroin use is specific to neurons. Pie charts showing the distribution of ATAC-seq peaks in neuronal cells from control subjects (A) and heroin users (B), as well as non-neuronal cells from control subjects (C) and heroin users (D). The number of promoter peaks was significantly increased in neurons (and to a smaller extent in non-neuronal cells) of heroin users compared to matched controls. (E) Venn diagram of ATAC peaks in neuronal populations from heroin users and controls. (F) Venn diagram of ATAC peaks in non-neuronal cells from heroin users and controls. UTR: untranslated region.

Supplementary Figure 3

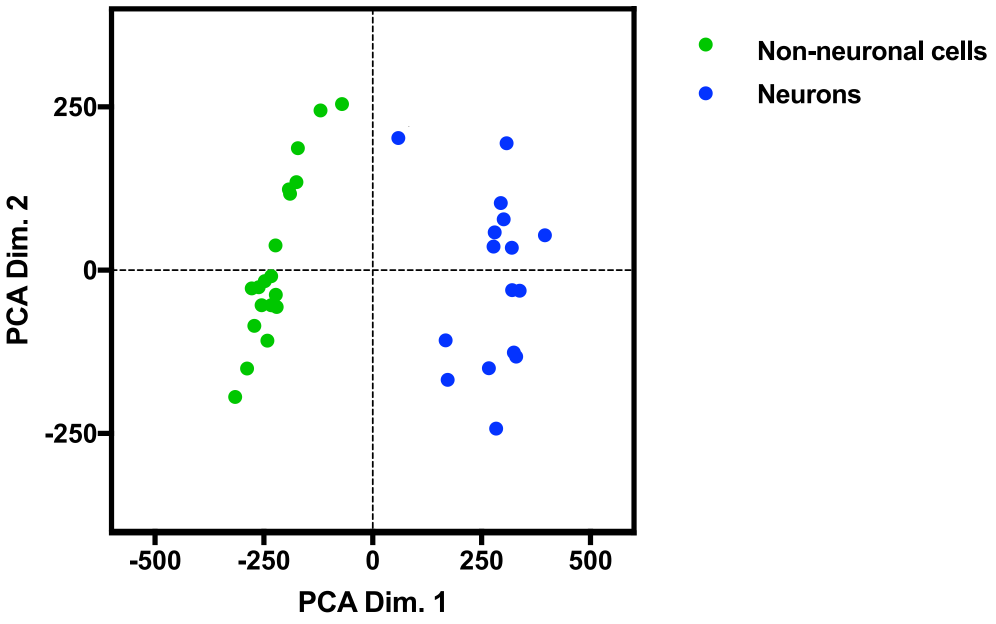

Supplementary Figure 3.
Marked differences of chromatin state between different cell populations. Principal component analysis (PCA) indicated strong separation of neuronal and non-neuronal cells based on ATAC-seq signals.

Supplementary Figure 4

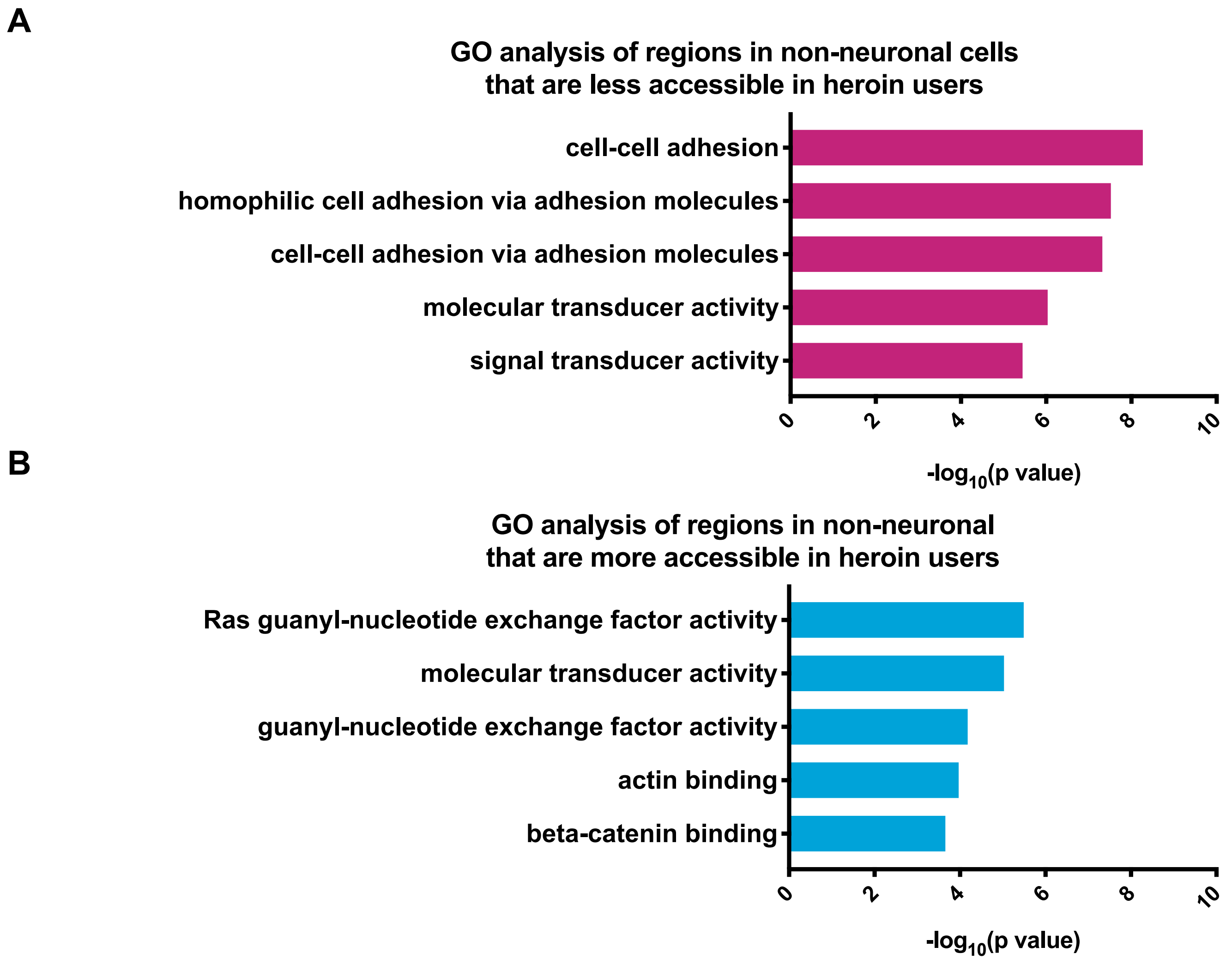

Supplementary Figure 4.
Gene ontology analysis of non-neuronal differential peaks. (A) Gene ontology (GO) analysis of less accessible peaks in heroin users revealed enrichment in cell adhesion and signal transduction (minimum hypergeometric test). (B) GO analysis of more accessible peaks in heroin users showed enrichment in nucleotide exchange, molecular transduction and actin binding (minimum hypergeometric test).

Supplementary Figure 5

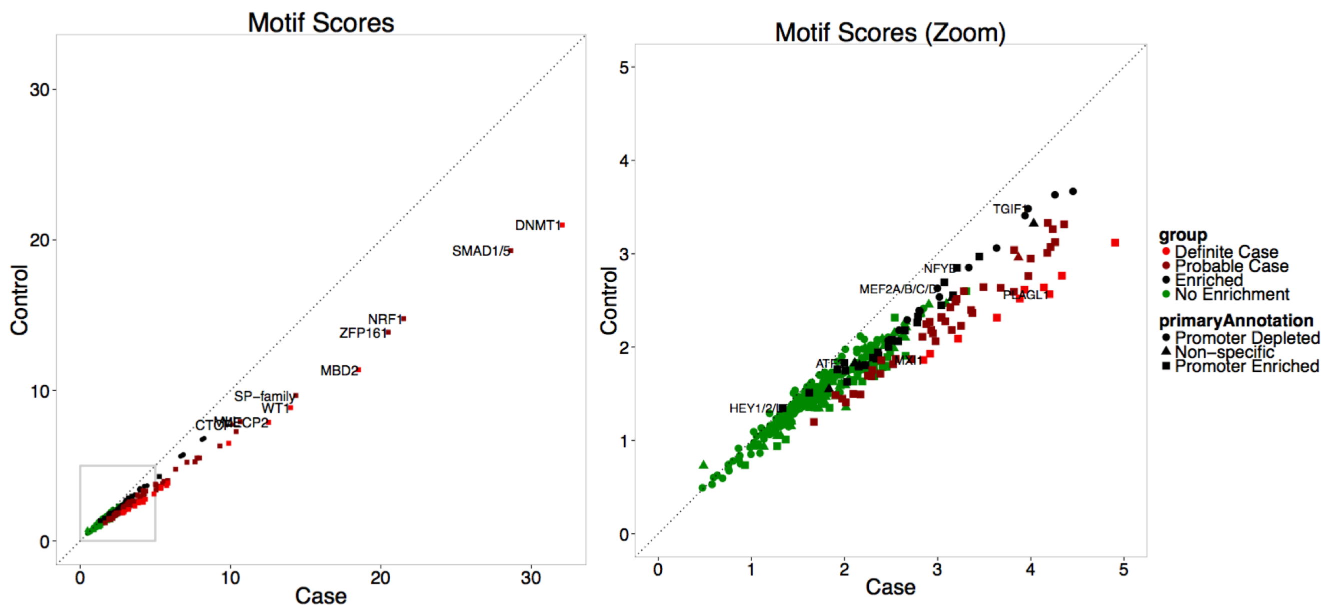

Supplementary Figure 5.
ATACseq footprinting analysis. The analysis revealed significant enrichment of transcription factors and DNA binding proteins (e.g. MBD2, MeCP2, DNMT1) in neurons of heroin users compared to controls.

Supplementary Figure 6

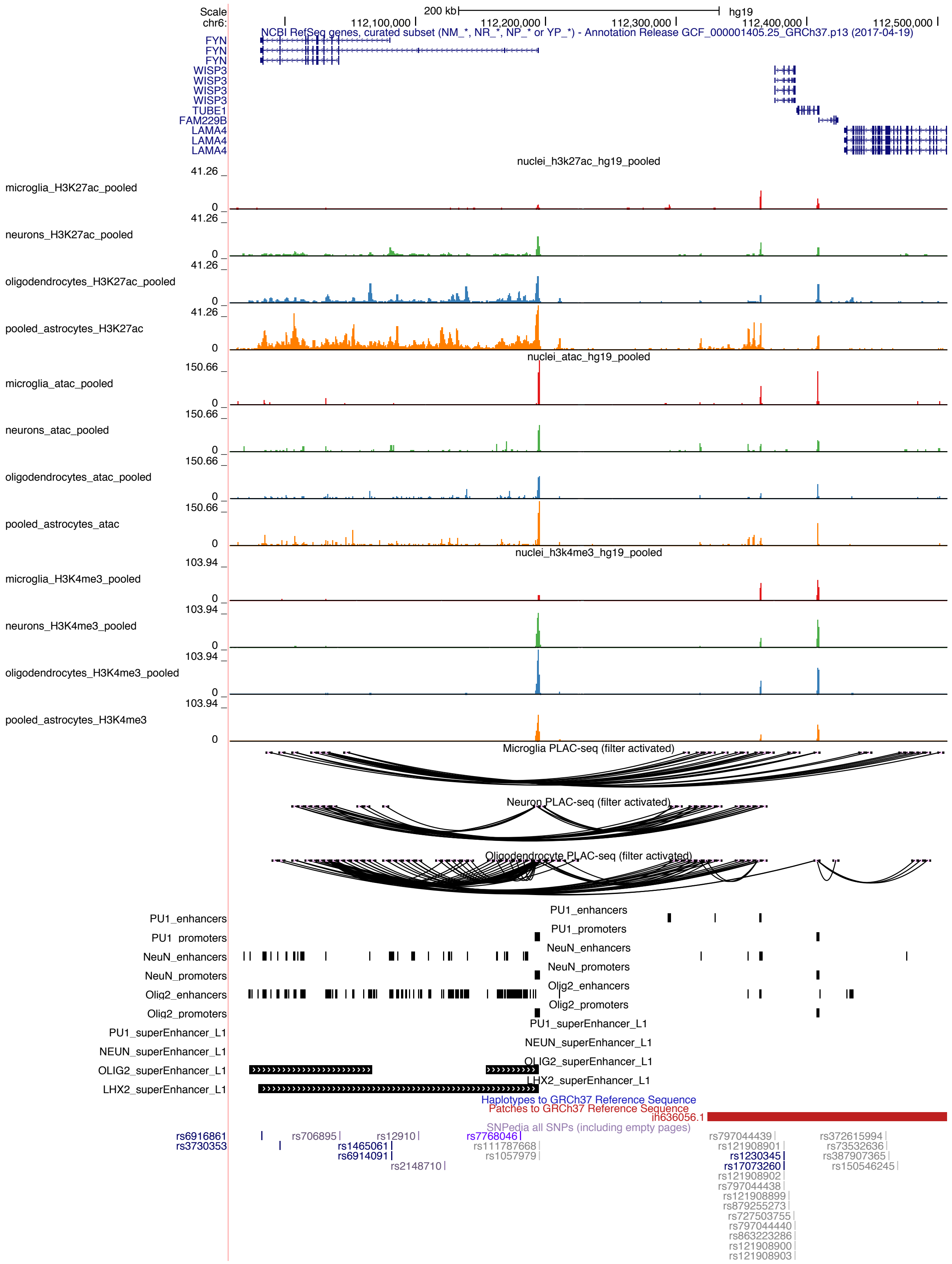

Supplementary Figure 6.
The putative regulatory region loops to the FYN promoter. Published brain PLACseq data[22] from neurons, microglia, astrocytes and oligodendrocytes showed looping between the putative regulatory region and several upstream and downstream promoters including that of *FYN*.

Supplementary Figure 7

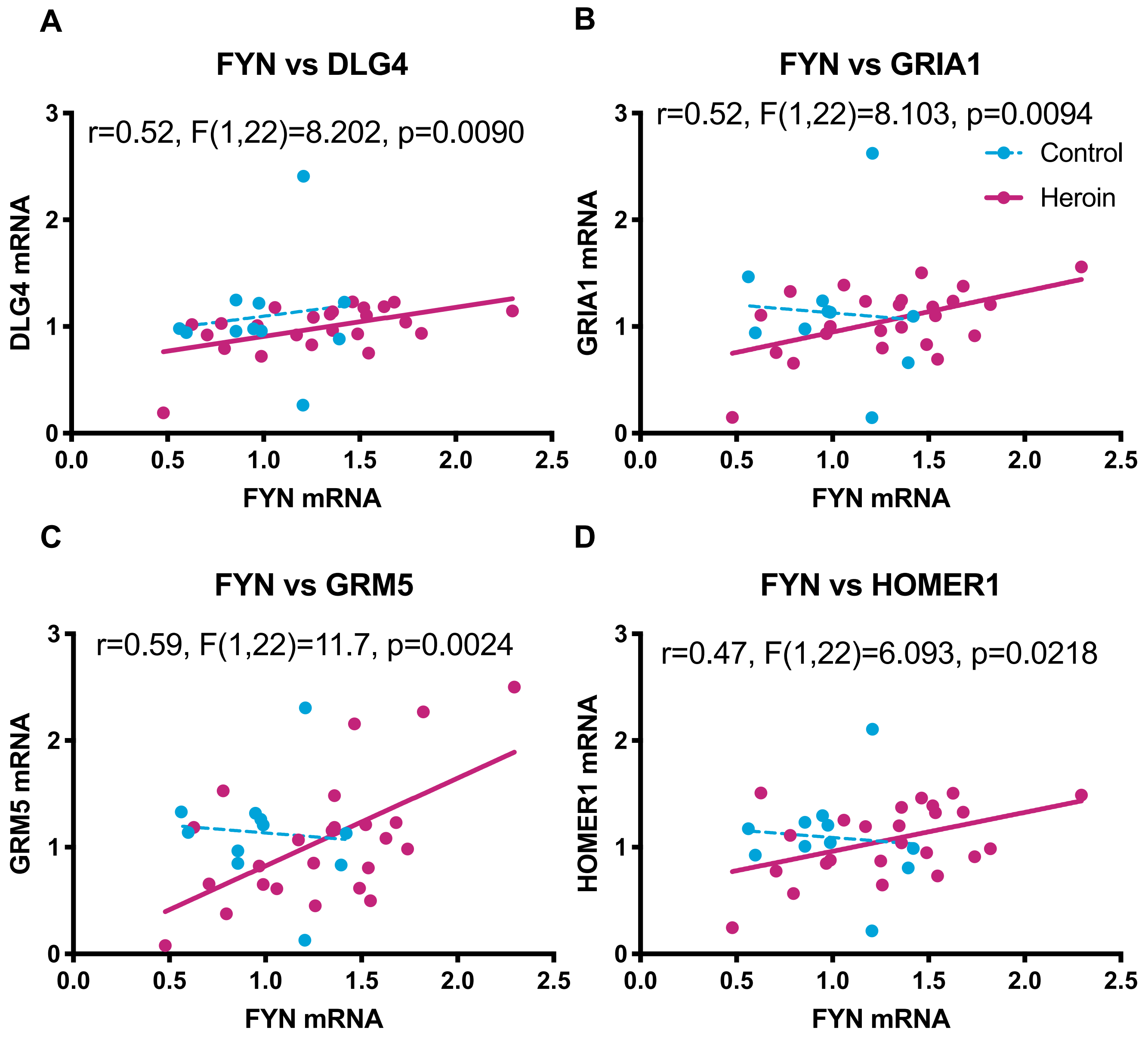

Supplementary Figure 7.

Differential correlations between *FYN* mRNA and post-synaptic density components. (A-D) Pearson correlations between normalized *FYN* mRNA levels and various members of the glutamatergic post-synaptic density in the putamen of human heroin users and matched controls. R, F and P values corresponding to linear regression in the heroin group are shown. *FYN* mRNA correlated positively with *DLG4* (A), *GRIA1* (B), *GRM5* (C) and *HOMER1* (D) mRNA in heroin users, but not in control subjects. Source data are provided as a Source Data file.

Supplementary Figure 8

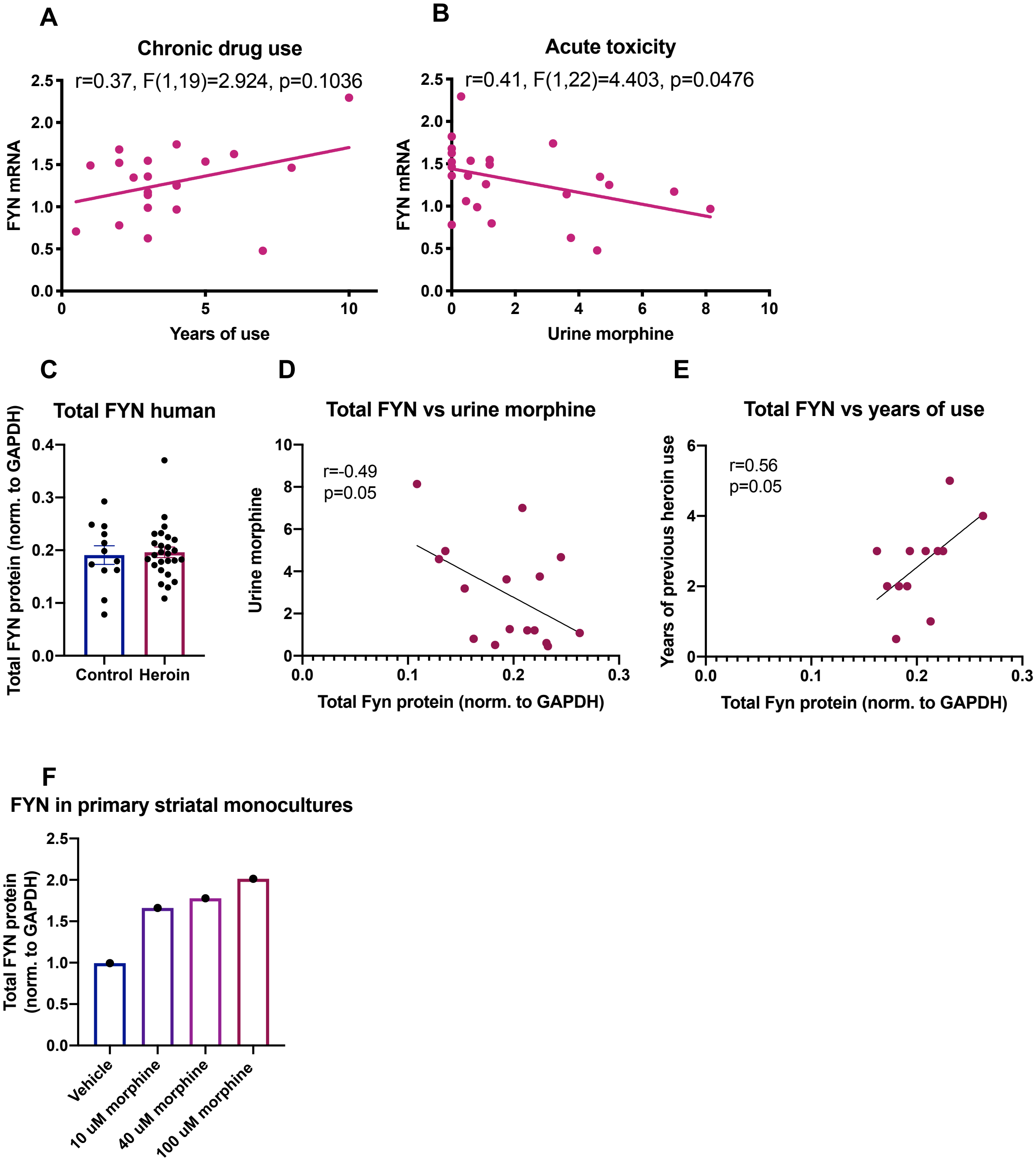

Supplementary Figure 8.

***FYN* expression is affected by drug use history and acute morphine toxicology**. (A,B) Pearson correlations between normalized FYN mRNA levels and years of previous drug use (A) and urine morphine [ng/ul] toxicology at the time of death (B). The data suggest an opposing effect of chronic heroin use and acute morphine use on *FYN* mRNA levels. (C) Total FYN protein levels in the post-mortem human putamen. There was no significant group difference between heroin users and controls (n=26 heroin users, n=12 controls, Student's t-test, t_{36}=0.2823, p=0.7793). (D) Pearson correlation between putamen FYN protein and urine morphine at the time of death revealed a significant negative correlation (linear regression, r=-0.49, $F_{1,14}$=4.479, p=0.05). (E) Pearson correlation between putamen FYN protein and years of previous heroin use revealed a significant positive correlation (linear regression, r=0.56, $F_{1,10}$=4.658, p=0.05). (F) Fyn protein tended to increase dose-dependently in primary striatal monocultures treated chronically with morphine. Data is represented as mean ± SEM. Source data are provided as a Source Data file.

Supplementary Figure 9

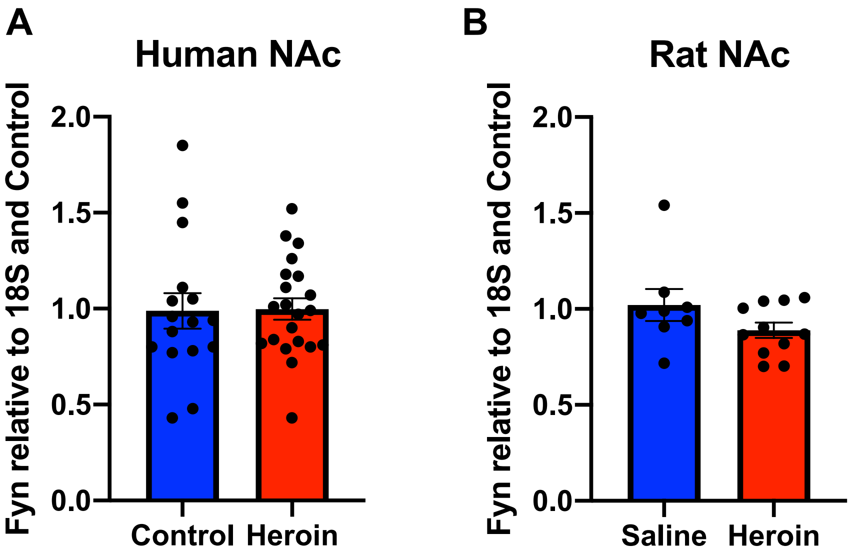

Supplementary Figure 9.

Transcriptional changes of FYN are specific to the dorsal striatum. *FYN* mRNA levels in the nucleus accumbens (NAc) of human heroin users (A; n=16/21 control/heroin, Student's t-test, t_{35}=0.09115, p=0.9279) and heroin self-administering rats (B; n=8/11 control/heroin, Student's t-test, t_{17}=1.55, p=0.1395). No group differences were observed in these brain regions. Data is represented as mean ± SEM. Source data are provided as a Source Data file.

Supplementary Figure 10

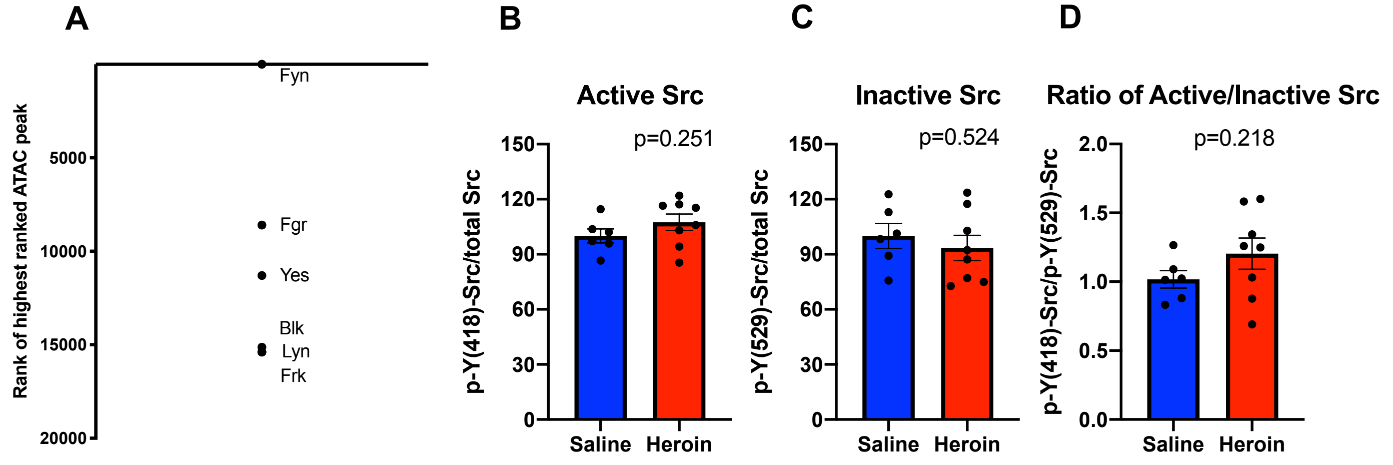

Supplementary Figure 10.

Other members of the SRC family of tyrosine kinases were not affected by heroin. (A) Ranks of the highest ranking ATAC peak assigned to genes encoding for SRC family members in the post-mortem human brain. Top 20,000 peaks are shown. FYN ranked #1, while only one other SRC family member (FGR) ranked within the top 10,000. (B) Active p(Y418)-Src was not changed in the dorsal striatum of heroin self-administering rats (n=6/8 control/heroin; Student's t-test, t_{12}=1.207, p=0.2508). (C) Inactive p(Y529)-Src was not changed in the dorsal striatum of heroin self-administering rats (n=6/8 control/heroin; Student's t-test, t_{12}=0.6566, p=0.5238). (D) The ratio of active/inactive p-Src is unchanged in the dorsal striatum of heroin self-administering rats (n=6/8 control/heroin, Student's t-test, t_{12}=1.3, p=0.218). Data is represented as mean ± SEM. Source data are provided as a Source Data file.

Supplementary Figure 11

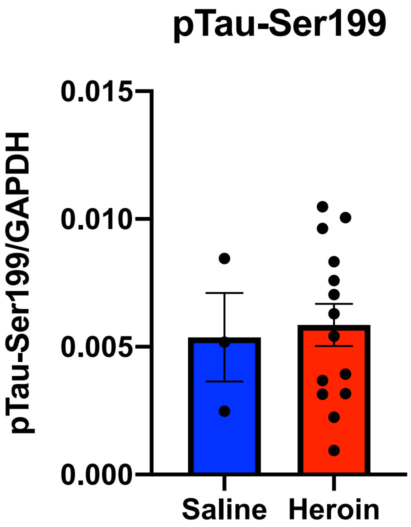

Supplementary Figure 11.
Heroin-induced Tau hyperphosphorylation is specific to residues targeted by FYN. Serine-199 phosphorylation was not changed in the caudate-putamen of heroin self-administering rats (n=3/14 control/heroin, Student's t-test, t_{15}=0.246, p=0.8090). Data is represented as mean ± SEM. Source data are provided as a Source Data file.

Supplementary Figure 12

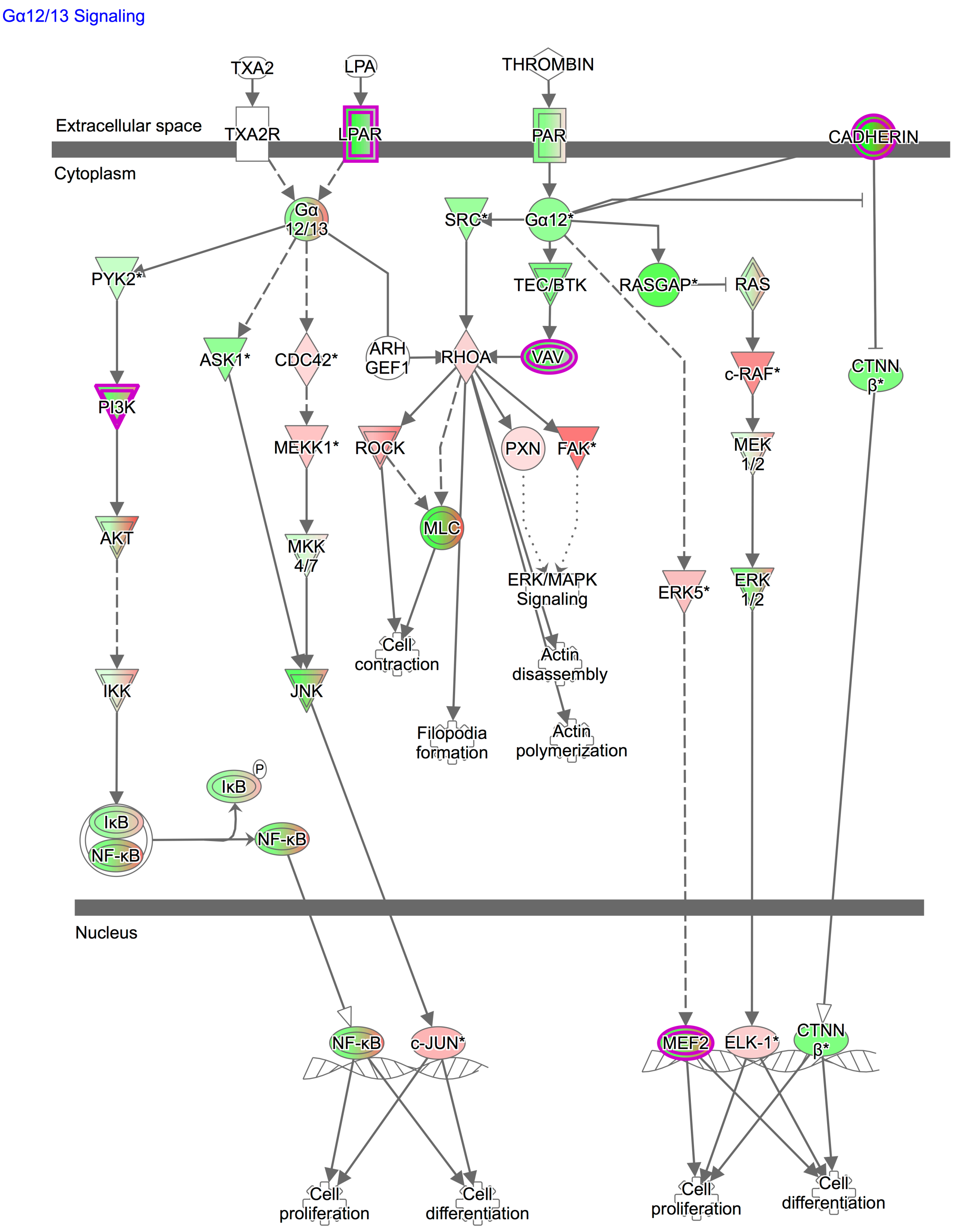

Supplementary Figure 12.
Network analysis. Ingenuity Pathway Analysis revealed focal adhesion kinase (FAK)-related signaling as one of the most significantly altered in heroin users.

Supplementary Figure 13

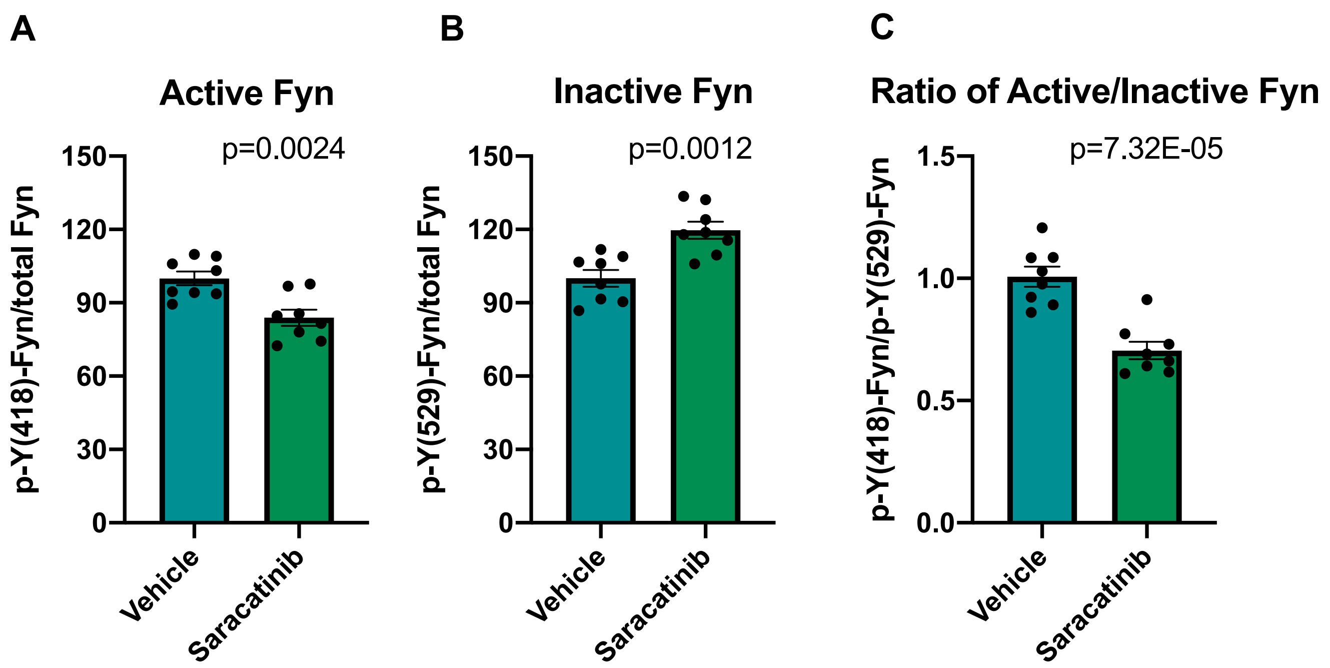

Supplementary Figure 13.
Intraperitoneal injection of saracatinib decreases dorsal striatal Fyn kinase activity. (A) Active p(Y418)-Fyn was decreased in the dorsal striatum of saracatinib-treated rats (5 mg/kg, n=8/group, Student's t-test, t_{14}=3.697, p=0.0024). (B) Inactive p(Y529)-Fyn was increased in the dorsal striatum of saracatinib-treated rats (5 mg/kg, n=8/group, Student's t-test, t_{14}=4.055, p=0.0012). (C) The ratio of active/inactive p-Fyn is significantly decreased in the dorsal striatum of saracatinib-treated rats (5 mg/kg n=8/group, Student's t-test, t_{14}=5.537, p=0.0001). Data is represented as mean ± SEM. Source data are provided as a Source Data file.

Supplementary Figure 14

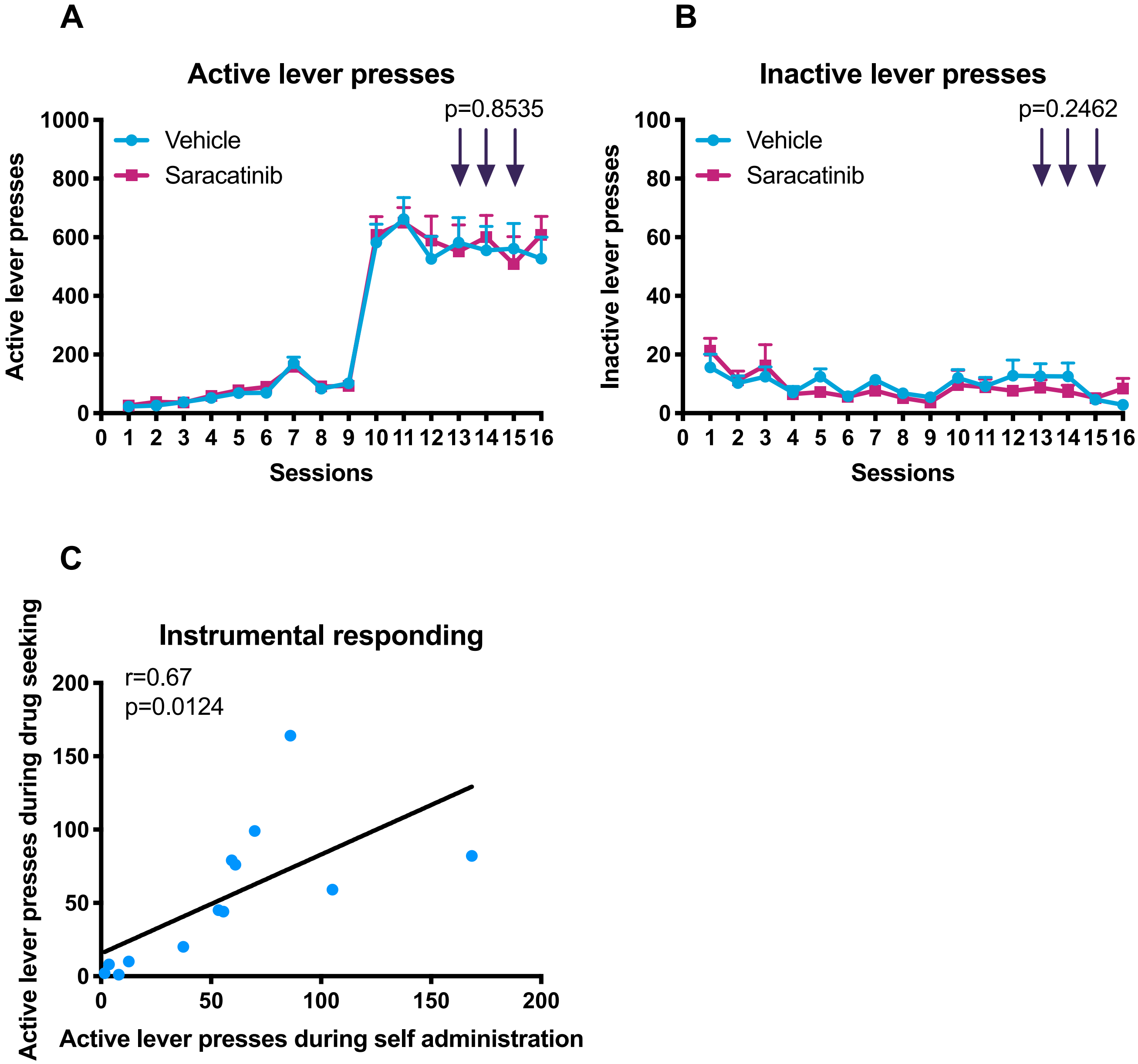

Supplementary Figure 14.

Saracatinib does not affect the self-administration of a palatable food reward. (A) Active lever presses during 1 hour long sucrose self-administration session in vehicle and saracatinib treated Long-Evans rats (n=11/group, two-way ANOVA, $F_{1,60}$=0.0344, p=0.8535). Arrows represent days of saracatinib administration. Data is represented as mean ± SEM. Two-way ANOVA revealed no significant group effect, indicating that saracatinib did not affect food self-administration. (B) Inactive lever presses during 3 hours long heroin self-administration session in vehicle and saracatinib treated Long-Evans rats (n=11/group, two-way ANOVA, $F_{1,60}$=1.371, p=0.2462). Arrows represent days of saracatinib administration. Data is represented as mean ± SEM. Inactive lever presses were not affected. (C) Saracatinib-induced decrease of active lever pressing during the three self-administration sessions when saracatinib was administered was predictive of the decreased level of responding during the cue-induced session (linear regression, r=0.67, $F_{1,11}$=8.919, p=0.0124). Data is represented as mean ± SEM. Source data are provided as a Source Data file.

Supplementary Figure 15

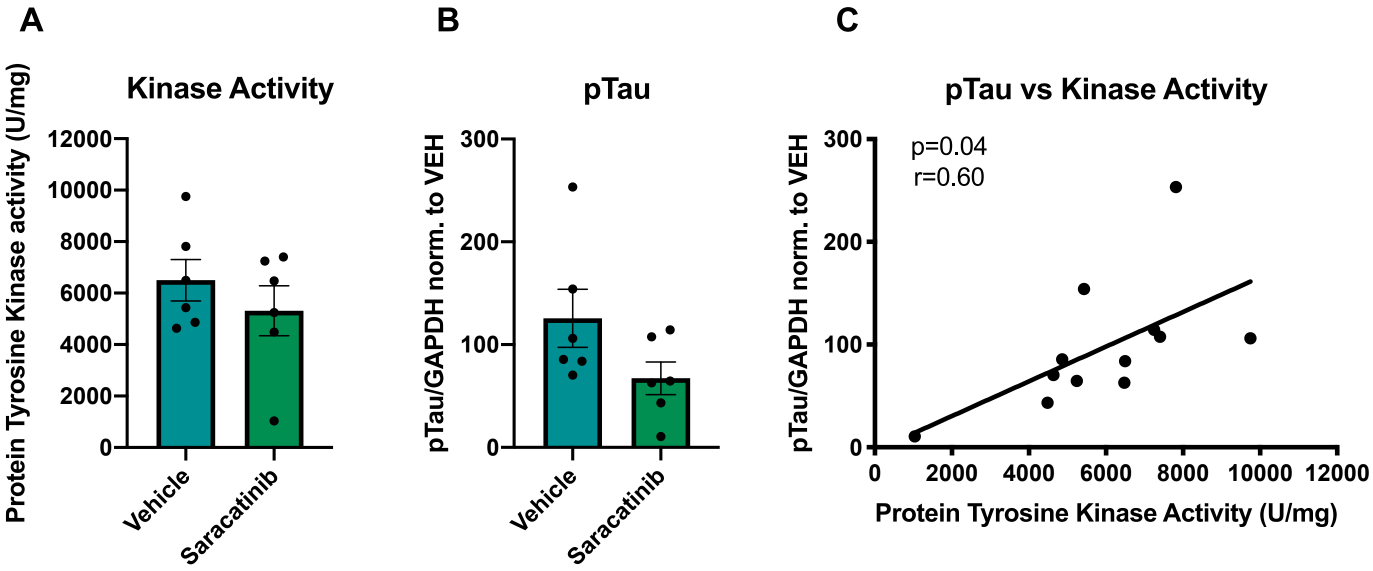

Supplementary Figure 15.

Saracatinib decreases dorsal striatal Fyn kinase activity and Tau phosphorylation. (A) Protein tyrosine kinase activity was slightly attenuated (n=6/group, Student's t-test, t_{10}=0.9368, p=0.37); in the dorsal striatum of heroin self-administering rats injected with vehicle or saracatinib for 3 consecutive days. (B) Tau phosphorylation tended to be decreased (n=6/group, Student's t-test, t_{10}=1.798, p=0.1024) in the dorsal striatum of heroin self-administering rats injected with vehicle or saracatinib for 3 consecutive days. (C) Pearson correlation between dorsal striatal protein tyrosine kinase activity and dorsal striatal Tau phosphorylation in heroin self-administering rats injected with vehicle or saracatinib for 3 consecutive days (linear regression, $F_{1,10}$=5.556, r=0.598; p=0.0401). The linear correlation suggests that saracatinib-induced decreases in tyrosine kinase activity might be related to diminished phosphorylation of the Fyn-target site on Tau protein. Data is represented as mean ± SEM. Source data are provided as a Source Data file.

Supplementary Figure 16

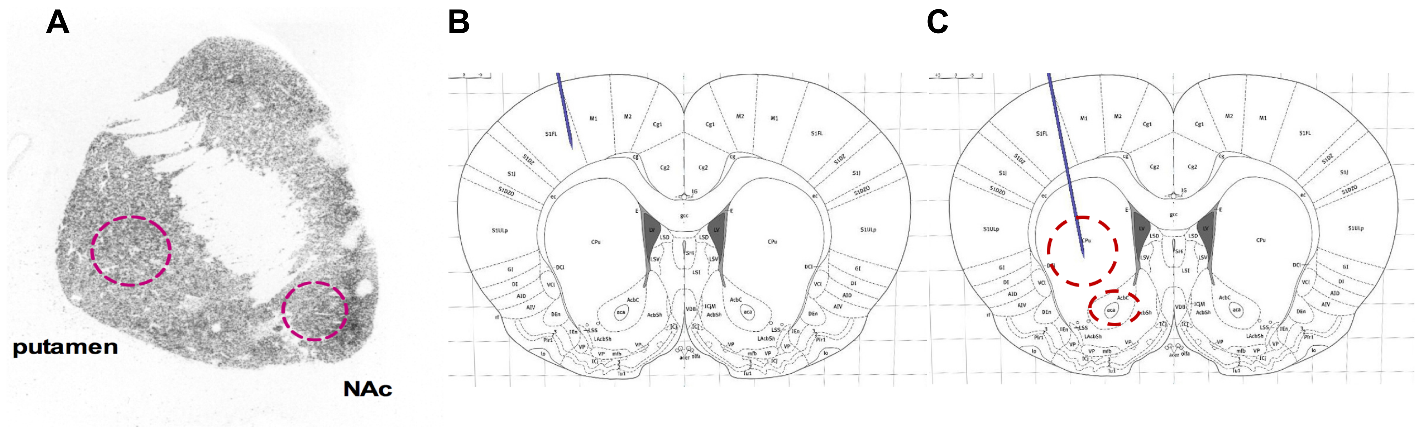

Supplementary Figure 16.

Schematic of brain regions assessed in this study. (A) Human nucleus accumbens (NAc) and putamen tissue was collected from the same coronal plane. (B) Guide cannulae were implanted at AP+1.7mm, ML+3.5mm and DV+3.5mm from bregma (C). For siRNA injections, needles were lowered through the guide cannulae to AP+1.7mm, ML+3.5mm and DV+5.2mm from bregma, and subsequently pulled back to AP+1.7mm, ML+3.5mm and DV+4.2mm from bregma. Red dotted circles show the areas of tissue collection for biochemical studies. We focused on the anterior, more medial part of the dorsal striatum, which is the area where long-lasting activation of Fyn has been previously described by Ron and colleagues during alcohol use.

Supplementary Figure 17

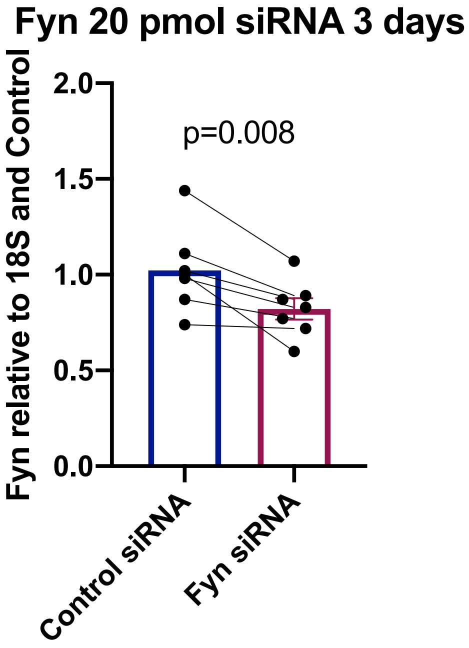

Supplementary Figure 17.
siRNA-mediated knock-down of Fyn decreases dorsal striatal Fyn mRNA levels. (A) *Fyn* mRNA was significantly decreased 3 days following the injection of 20 pmol FYN siRNA (n=7 rats, Student's t-test, t_6=3.833, p=0.0087). Data is represented as mean ± SEM. Source data are provided as a Source Data file.

Supplementary Figure 18

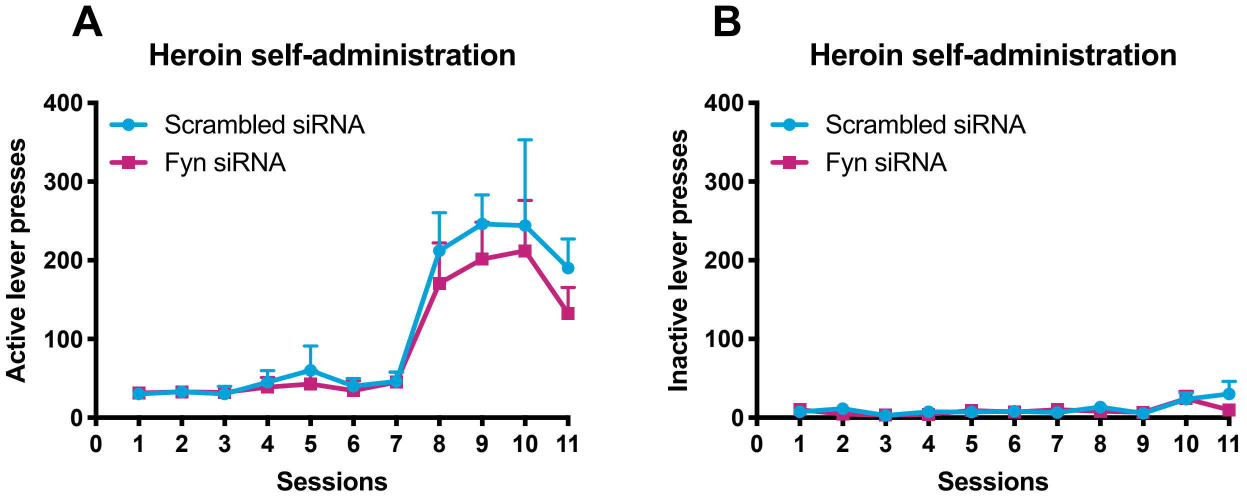

Supplementary Figure 18.
Heroin self-administration data from siRNA-injected cohort. (A) active lever presses and (B) inactive lever presses during heroin self-administrating training prior to siRNA injection. Following training, animals were injected with 20 pmol FYN siRNA and subsequently underwent cue- or heroin-induced sessions (Figure 6E-H). N=5 rats/group, data is represented as mean ± SEM. Source data are provided as a Source Data file.

Supplementary Figure 19

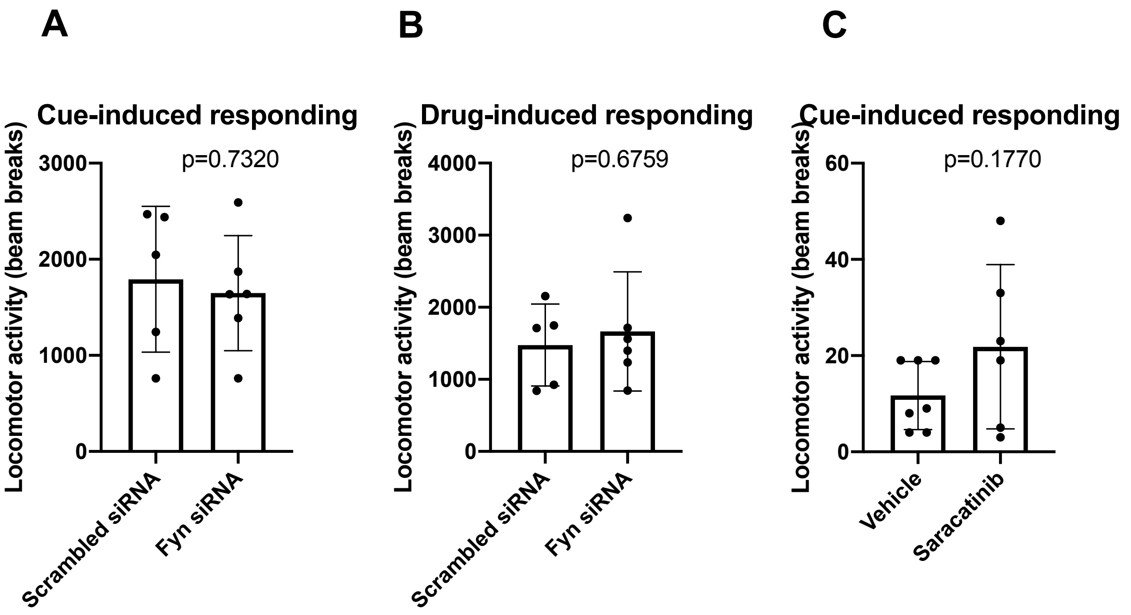

Supplementary Figure 19.

Inhibition of Fyn in the dorsal striatum does not affect general locomotor activity. (A) Locomotor activity during cue-induced responding was not affected in siRNA-injected rats (n=5/6 scrambled/Fyn, Student's t-test, t_9=0.3532, p=0.732). (B) Locomotor activity during cue-induced responding was not affected in siRNA-injected rats (n=5/6 scrambled/Fyn, Student's t-test, t_9=0.432, p=0.6759). (C) Locomotor activity during cue-induced responding was not affected in saracatinib injected rats (n=7/6 vehicle/saracatinib, Student's t-test, t_{11}=1.439, p=0.1779). Data is represented as mean ± SEM. Source data are provided as a Source Data file.

Supplementary Figure 20

Fig4F

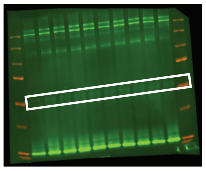

Layout: (Gel A)
1- Licor Ladder
2- 1 (Heroin)
3- 2 (Saline)
4- 3 (Heroin)
5- 4 (Heroin)
6- 5 (Saline)
7- 6 (Saline)
8- 7 (Heroin)
9- 8 (Heroin)
10- 9 (Heroin)
11- 10 (Saline)
12- Licor Ladder

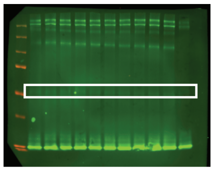

Layout: (Gel B)
1- Licor Ladder
2- 11 (Heroin)
3- 12 (Saline)
4- 13 (Heroin)
5- 14 (Saline)
6- 15 (Saline)
7- 16 (Heroin)
8- 17 (Heroin)
9- 18 (Saline)
10- 19 (Heroin)
11- 20 (Heroin)
12- 19 (IgG control)

Fig4G

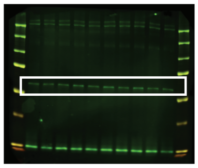

Layout: (Gel A)
1- Licor Ladder
2- 1 (Heroin)
3- 2 (Saline)
4- 3 (Heroin)
5- 4 (Heroin)
6- 5 (Saline)
7- 6 (Saline)
8- 7 (Heroin)
9- 8 (Heroin)
10- 9 (Heroin)
11- 10 (Saline)
12- Licor Ladder

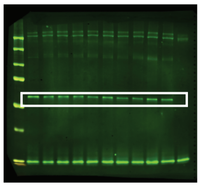

Layout: (Gel B)
1- Licor Ladder
2- 11 (Heroin)
3- 12 (Saline)
4- 13 (Heroin)
5- 14 (Saline)
6- 15 (Saline)
7- 16 (Heroin)
8- 17 (Heroin)
9- 18 (Saline)
10- 19 (Heroin)
11- 20 (Heroin)
12- 19 (IgG control)

Fig4K

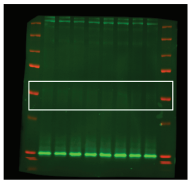

Layout: (Gel A)
1- Licor Ladder
2- 5 (Heroin)
3- 25 (Saline)
4- 8 (Heroin)
5- 26 (Saline)
6- 9 (Heroin)
7- 27 (Saline)
8- 12 (Heroin)
9- 28 (Saline)
10- Licor Ladder

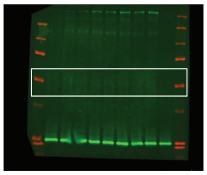

Layout: (Gel B)
1- Licor Ladder
2- 18 (Heroin)
3- 29 (Saline)
4- 20 (Heroin)
5- 30 (Saline)
6- 21 (Heroin)
7- 31 (Saline)
8- 22 (Heroin)
9- 32 (Saline)
10- 30 (IgG control)
11- Licor Ladder

Fig4L

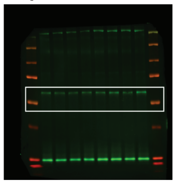

Layout: (Gel C)
1- Licor Ladder
2- 5 (Heroin)
3- 25 (Saline)
4- 8 (Heroin)
5- 26 (Saline)
6- 9 (Heroin)
7- 27 (Saline)
8- 12 (Heroin)
9- 28 (Saline)
10- Licor Ladder

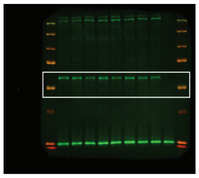

Layout: (Gel D)
1- Licor Ladder
2- 18 (Heroin)
3- 29 (Saline)
4- 20 (Heroin)
5- 30 (Saline)
6- 21 (Heroin)
7- 31 (Saline)
8- 22 (Heroin)
9- 32 (Saline)
10- 30 (IgG control)
11- Licor Ladder

Fig4O

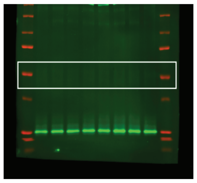

Layout: (Gel A)
1- Licor Ladder
2- 51 (Heroin)
3- 52 (Saline)
4- 53 (Heroin)
5- 54 (Saline)
6- 55 (Heroin)
7- 56 (Saline)
8- 57 (Heroin)
9- 58 (Saline)
10- Licor Ladder

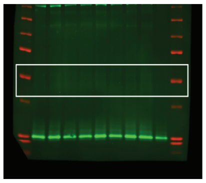

Layout: (Gel B)
1- Licor Ladder
2- 59 (Heroin)
3- 60 (Saline)
4- 61 (Heroin)
5- 62 (Saline)
6- 63 (Heroin)
7- 64 (Saline)
8- 65 (Heroin)
9- 66 (Saline)
10- 59 (IgG control)
11- Licor Ladder

Fig4P

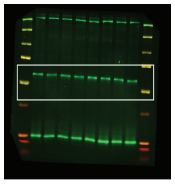

Layout: (Gel C)
1- Licor Ladder
2- 51 (Heroin)
3- 52 (Saline)
4- 53 (Heroin)
5- 54 (Saline)
6- 55 (Heroin)
7- 56 (Saline)
8- 57 (Heroin)
9- 58 (Saline)
10- Licor Ladder

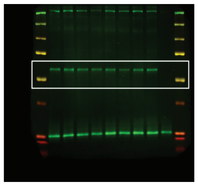

Layout: (Gel D)
1- Licor Ladder
2- 59 (Heroin)
3- 60 (Saline)
4- 61 (Heroin)
5- 62 (Saline)
6- 63 (Heroin)
7- 64 (Saline)
8- 65 (Heroin)
9- 66 (Saline)
10- 59 (IgG control)
11- Licor Ladder

Fig5B

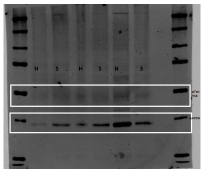

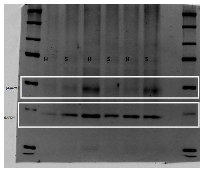

Fig5C

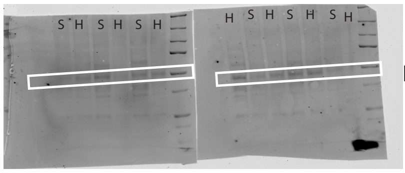

pTau

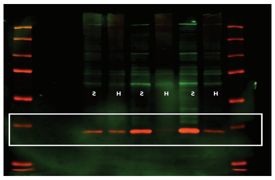

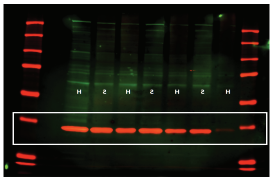

GAPDH (also stained for BRD4)

Ext. Data Fig 8C-E

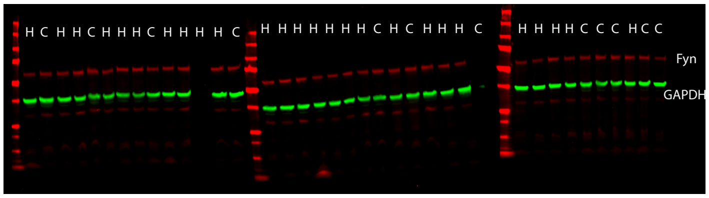

Ext. Data Fig 8F

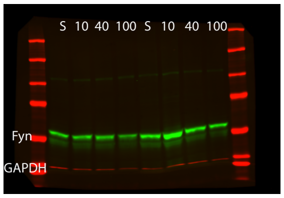

Ext. Data Fig 10B

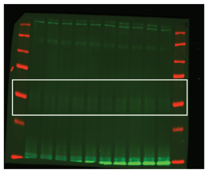

Layout: (Gel A)
1- Licor Ladder
2- 2 (Saline)
3- 3 (Heroin)
4- 4 (Heroin)
5- 5 (Saline)
6- 6 (Saline)
7- 8 (Heroin)
8- 9 (Heroin)
9- 12 (Saline)
10- 13 (Heroin)
11- 15 (Saline)
12- Licor Ladder

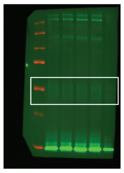

Layout: (Gel B)
1- Licor Ladder
2- 16 (Heroin)
3- 17 (Heroin)
4- 18 (Saline)
5- 19 (Heroin)
6- 19 (IgG control)

Ext. Data Fig 10C

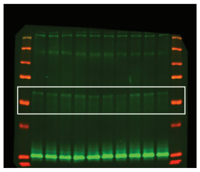

Layout: (Gel C)
1- Licor Ladder
2- 2 (Saline)
3- 3 (Heroin)
4- 4 (Heroin)
5- 5 (Saline)
6- 6 (Saline)
7- 8 (Heroin)
8- 9 (Heroin)
9- 12 (Saline)
10- 13 (Heroin)
11- 15 (Saline)
12- Licor Ladder

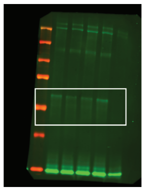

Layout: (Gel D)
1- Licor Ladder
2- 16 (Heroin)
3- 17 (Heroin)
4- 18 (Saline)
5- 19 (Heroin)
6- 19 (IgG control)

Supplementary Figures 20 and 21 show all uncropped Western blots used in this study. Molecular weight marker is defined in Supplementary Figure 21.

Supplementary Figure 21

Ext. Data Fig 11

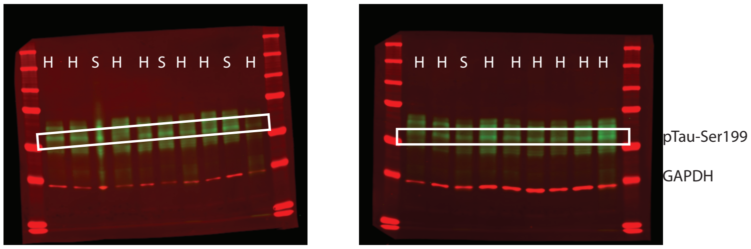

Ext. Data Fig 13A

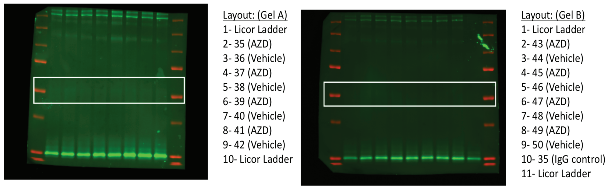

Ext. Data Fig 13B

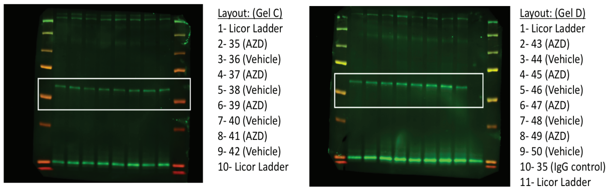

Ext. Data Fig 15B

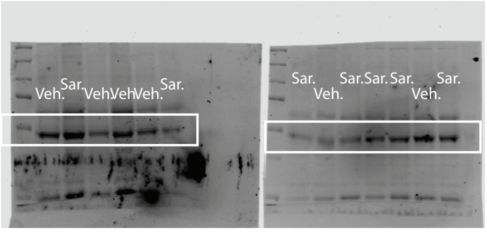

Molecular weight marker used (LICOR 928-40000)

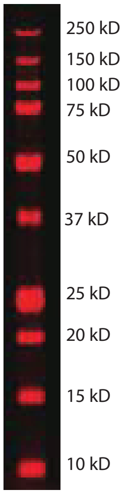

Supplementary Figures 20 and 21 show all uncropped Western blots used in this study. Molecular weight marker is defined in Supplementary Figure 21.
