# Supplementary material for: Chromatin accessibility mapping of the striatum identifies tyrosine kinase FYN as a therapeutic target for heroin use disorder
Source: Nat Commun. 2020 Sep 14;11:4634. doi: 10.1038/s41467-020-18114-3 (PMC7490718; doi:10.1038/s41467-020-18114-3)
Supplement: Supplementary file 3 — Description of Additional Supplementary Information [file 41467_2020_18114_MOESM3_ESM.pdf]

Description of Additional Supplementary Files

Supplementary Data 1. Demographic information for human heroin users and control subjects included in the study.

Supplementary Data 2. Quality control metrics of the human ATAC libraries. ChrM: mitochondrial chromosomes, PBC: PCR bottleneck coefficient, NSC: normalized strand cross-correlation coefficient, RSC: relative strand cross-correlation coefficient. FRiP: fraction of reads in peaks.

Supplementary Data 3. List of accessible regions in neurons, ranked by proportion of variance explained by disease. P values from quasi-likelihood F-test, corrected using false discovery rate estimation.

Supplementary Data 4. List of accessible regions in non-neuronal cells, ranked by proportion of variance explained by disease. P values from quasi-likelihood F-test, corrected using false discovery rate estimation.

Supplementary Data 5. Epigenetic marks (Roadmap) showing significant enrichment at ATAC peaks specific to neurons from heroin users. P values from Fisher's exact test, Benjamini-Hochberg correction.

Supplementary Data 6. Epigenetic marks (Roadmap) showing significant enrichment at ATAC peaks specific to neurons from control subjects. P values from Fisher's exact test, Benjamini-Hochberg correction.

Supplementary Data 7. Epigenetic marks (Roadmap) showing significant enrichment at ATAC peaks specific to non-neuronal cells from heroin users. P values from Fisher's exact test, Benjamini-Hochberg correction.

Supplementary Data 8. Epigenetic marks (Roadmap) showing significant enrichment at ATAC peaks specific to non-neuronal cells from control subjects. P values from Fisher's exact test, Benjamini-Hochberg correction.

Supplementary Data 9. Enrichment analysis of ATAC peaks with genomic regions in neurons from heroin users. P values from Fisher's exact test, Benjamini-Hochberg correction.

Supplementary Data 10. Enrichment analysis of ATAC peaks with genomic regions in neurons from control subjects. P values from Fisher's exact test, Benjamini-Hochberg correction.

Supplementary Data 11. Enrichment analysis of ATAC peaks with genomic regions in non-neuronal cells from heroin users. P values from Fisher's exact test, Benjamini-Hochberg correction.

Supplementary Data 12. Enrichment analysis of ATAC peaks with genomic regions in non-neuronal cells from control subjects. P values from Fisher's exact test, Benjamini-Hochberg correction.

Supplementary Data 13. Enrichment of histone marks from human brain ENCODE data over the putative FYN regulatory locus identified in the variance analysis. Nominal signal P values from ENCODE are shown.

Supplementary Data 14. Heroin-induced disregulation of Src family members in publicly available datasets from GEO. P values calculated with limma, Benjamini-Hochberg correction.
