# Supplementary material for: Chromatin accessibility mapping of the striatum identifies tyrosine kinase FYN as a therapeutic target for heroin use disorder
Source: Nat Commun. 2020 Sep 14;11:4634. doi: 10.1038/s41467-020-18114-3 (PMC7490718; doi:10.1038/s41467-020-18114-3)
Supplement: Supplementary file 18 — Reporting Summary [file 41467_2020_18114_MOESM18_ESM.pdf]

nature research

Corresponding author(s): Yasmin L. Hurd

Last updated by author(s): Jun 29, 2020

Reporting Summary

Nature Research wishes to improve the reproducibility of the work that we publish. This form provides structure for consistency and transparency in reporting. For further information on Nature Research policies, see our Editorial Policies and the Editorial Policy Checklist.

Statistics

For all statistical analyses, confirm that the following items are present in the figure legend, table legend, main text, or Methods section.

n/a	Confirmed	
☐	☒	The exact sample size (n) for each experimental group/condition, given as a discrete number and unit of measurement
☐	☒	A statement on whether measurements were taken from distinct samples or whether the same sample was measured repeatedly
☐	☒	The statistical test(s) used AND whether they are one- or two-sided<br>*Only common tests should be described solely by name; describe more complex techniques in the Methods section.*
☐	☒	A description of all covariates tested
☐	☒	A description of any assumptions or corrections, such as tests of normality and adjustment for multiple comparisons
☐	☒	A full description of the statistical parameters including central tendency (e.g. means) or other basic estimates (e.g. regression coefficient) AND variation (e.g. standard deviation) or associated estimates of uncertainty (e.g. confidence intervals)
☐	☒	For null hypothesis testing, the test statistic (e.g. F, t, r) with confidence intervals, effect sizes, degrees of freedom and P value noted<br>*Give P values as exact values whenever suitable.*
☒	☐	For Bayesian analysis, information on the choice of priors and Markov chain Monte Carlo settings
☒	☐	For hierarchical and complex designs, identification of the appropriate level for tests and full reporting of outcomes
☐	☒	Estimates of effect sizes (e.g. Cohen's d, Pearson's r), indicating how they were calculated

Our web collection on statistics for biologists contains articles on many of the points above.

Software and code

Policy information about availability of computer code

Data collection	No software was used for data collection.
Data analysis	The following software was used for statistical analysis: R 3.6.2., JMP10, Graphpad Prism 8, STAR aligner 2.5.0, MACS v2.1, Fiji 2017, MATLAB9.7, Primer3 version 0.4.0.

For manuscripts utilizing custom algorithms or software that are central to the research but not yet described in published literature, software must be made available to editors and reviewers. We strongly encourage code deposition in a community repository (e.g. GitHub). See the Nature Research guidelines for submitting code & software for further information.

Data

Policy information about availability of data

All manuscripts must include a data availability statement. This statement should provide the following information, where applicable:

- Accession codes, unique identifiers, or web links for publicly available datasets
- A list of figures that have associated raw data
- A description of any restrictions on data availability

The sequencing data are available in GEO under accession code GSE136042. Uncropped and unprocessed gels are included in Supplementary Figures 20 and 21. Quantification of gels, PCRs, luciferase assays, immunocytochemistry, behavioral assays and all other relevant data (associated with Figures 2-6, Supplementary Figures 2, 4, 7-11, 13-15, and 17-19) are available in the Source Data. Genomic regions were collected from the following data sources. Transcription factor binding sites (TFBS) were downloaded from ENCODE [https://www.encodeproject.org], CODEX [http://codex.stemcells.cam.ac.uk], Cistrome [http://cistrome.org/Cistrome/Cistrome_Project.html]. Enhancer segments and repressed segments were downloaded from ENCODE. Tissue clustered DNase hypersensitive sites were downloaded from Sheffield et al [http://dnase.genome.duke.edu]. CpG islands, microsatellites, genome repeat regions etc. were obtained from UCSC Genome Browser database. Transcriptional factor motifs were obtained from JASPAR [http://jaspar.genereg.net]. Epigenomics modification regions of various conditions

were downloaded from Cistrome.

Field-specific reporting

Please select the one below that is the best fit for your research. If you are not sure, read the appropriate sections before making your selection.

[x] Life sciences [ ] Behavioural & social sciences [ ] Ecological, evolutionary & environmental sciences

For a reference copy of the document with all sections, see nature.com/documents/nr-reporting-summary-flat.pdf

Life sciences study design

All studies must disclose on these points even when the disclosure is negative.

Sample size	Sample sizes were determined based on previous experience in our group and the literature. (e.g. similar experiments we performed in Egervari et al, Biol Psychiatry 2017; Miller et al, Mol Psychiatry 2019)
Data exclusions	No data was excluded.
Replication	In vivo experiments were performed in at least 5 biological replicates. In vitro experiments were performed in three biological replicates. Replication attempts were successful.
Randomization	Samples were assigned randomly, wherever applicable.
Blinding	ATACseq libraries were prepared by a blinded investigator. Quantification of molecular experiments (immunocytochemistry, Western blots, luciferase assays) was performed by blinded investigators.

Reporting for specific materials, systems and methods

We require information from authors about some types of materials, experimental systems and methods used in many studies. Here, indicate whether each material, system or method listed is relevant to your study. If you are not sure if a list item applies to your research, read the appropriate section before selecting a response.

Materials & experimental systems

n/a	Involved in the study	
[ ]	[x]	Antibodies
[ ]	[x]	Eukaryotic cell lines
[x]	[ ]	Palaeontology and archaeology
[ ]	[x]	Animals and other organisms
[ ]	[x]	Human research participants
[x]	[ ]	Clinical data
[x]	[ ]	Dual use research of concern

Methods

n/a	Involved in the study	
[x]	[ ]	ChIP-seq
[x]	[ ]	Flow cytometry
[x]	[ ]	MRI-based neuroimaging

Antibodies

Antibodies used	Antibodies used in this study were: pTau-Y18 (GTX54658, GeneTex), pTau-Ser199 (44734G, Thermo Fisher), DARPP-32 (2306S, Cell Signaling), pY(418)-Src (ab4816, Abcam), pY(529)-Src (ab32078, Abcam), FYN (4023S, Cell Signaling), SRC (ab16885, Abcam), NeuN Alexa488-conjugated (MAB377X, Millipore), GAPDH (MAB374, Millipore), AlexaFluor-488 secondary antibodies (A32732 and A32723), LICOR goat anti-rabbit IRDye 680 (LICOR, 926-68071), LICOR goat anti-mouse IRDye 800 (LICOR, 926-32210). Dilutions for all antibodies are provided in the Methods.
Validation	Primary antibodies were validated by the manufacturer as follows:<br>1. pTau-Y18 (GTX54658, GeneTex): validated for human, mouse, rat, for Western blot, ICC, IHC and IF<br>2. pTau-Ser199 (44734G, Thermo Fisher): validated for human, mouse, rat, for Western blot, ICC, IHC and IF<br>3. DARPP-32 (2306S, Cell Signaling): validated for mouse and rat, for Western blot, ICC, IHC and IF<br>4. pY(418)-Src (ab4816, Abcam): validated for mouse, chicken, human rat, for Western blot and IHC (frozen and paraffin-embedded), validated for IP-Westerns as performed here by e.g. Gibb et al, J Neurochem 2011<br>5. pY(529)-Src (ab32078, Abcam): validated for mouse human rat, for Western blot, IHC, ICC, IF, IP, dot blot, validated for IP-Westerns as performed here by e.g. Gibb et al, J Neurochem 2011<br>6. FYN (4023S, Cell Signaling):validated for mouse, human, rat, for Western blot<br>7. SRC (ab16885, Abcam): validated for mouse, human, rat, for Western blot, IP, flow cytometry<br>8. NeuN Alexa488-conjugated (MAB377X, Millipore): validated for mouse, human, rat, for IHC, validated for flow cytometry as performed here by e.g. Fullard et al, Genome Res 2018<br>9. GAPDH (MAB374, Millipore): validated for mouse, human, rat, chicken, rabbit and others, for Western blot, ICC, IHC, IF, IP, ELISA

Eukaryotic cell lines

Policy information about cell lines

Cell line source(s)	HEK293T cells were obtained from ATCC.
Authentication	No additional authentication was performed.
Mycoplasma contamination	Line tested negative for mycoplasma contamination.
Commonly misidentified lines (See ICLAC register)	None used.

Animals and other organisms

Policy information about studies involving animals; ARRIVE guidelines recommended for reporting animal research

Laboratory animals	Adult male Long-Evans rats (approximately 2-3 months old) were used.
Wild animals	This study did not involve the use of wild animals.
Field-collected samples	This study did not involved the use of field-collected samples.
Ethics oversight	Protocols were approved by Icahn School of Medicine's review board.

Note that full information on the approval of the study protocol must also be provided in the manuscript.

Human research participants

Policy information about studies involving human research participants

Population characteristics	Human brains from a homogeneous cohort of Caucasian subjects from apparent heroin overdose and normal controls (determined by self-report and ancestral informative marker analysis)were collected at autopsy within 24 hours of time of death. Detailed demographic information is available in Supplementary Data 1.
Recruitment	Samples were obtained from the Department of Forensic and Insurance Medicine, Semmelweis University, Hungary. Informed consent from legally authorized representatives was obtained at the time of autopsy
Ethics oversight	Protocols were approved by Semmelweis University's and Icahn School of Medicine's review boards.

Note that full information on the approval of the study protocol must also be provided in the manuscript.
